# Case Report: Treatment of Femoral Non-union With Rib and Iliac Crest Autografts and rhBMP-2 in a Cat

**DOI:** 10.3389/fvets.2021.756167

**Published:** 2021-11-25

**Authors:** Cheng-Shu Chung, Lee-Shuan Lin, Yi-Min Teo

**Affiliations:** ^1^Laboratory of Veterinary Surgery, Department of Veterinary Medicine, College of Veterinary Medicine, National Pingtung University of Science and Technology, Pingtung, Taiwan; ^2^Laboratory of Veterinary Diagnostic Imaging, Department of Veterinary Medicine, College of Veterinary Medicine, National Pingtung University of Science and Technology, Pingtung, Taiwan; ^3^Division of Small Animal Surgery, Veterinary Medical Teaching Hospital, National Pingtung University of Science and Technology, Pingtung, Taiwan

**Keywords:** cat, rhBMP-2, rib autograft, iliac crest autograft, non-union

## Abstract

A 5-year-old, intact male Bengal cat weighing 5.2 kg was referred for the fixation failure of a right femoral fracture. Multiple surgical revisions failed, and atrophic non-union was diagnosed. The cat was then admitted for a final revision surgery using locking plate fixation in conjunction with rib and iliac crest autografts and recombinant human bone morphogenetic protein 2 (rhBMP-2). The fracture site was debrided and stabilized before filling the defect with 1.8 cm of rib bone autograft. The residual space in the defect was then filled with an iliac crest autograft. Finally, a 3 ×5 cm absorbable collagen sponge soaked with 0.5 mL of 0.2 mg/mL rhBMP-2 solution was placed around the defect. No significant complications were noted postoperatively. Bone healing was noted 2 months postoperatively, and it continued for 12 months. Although mild lameness remained, the cat's ambulatory function and quality of life were good. To the authors' knowledge, this is the first case report of a clinical transplantation of a rib segment as an autograft in combination with rhBMP-2 in a cat with a large bone defect.

## Introduction

Fracture non-union, due to mechanical or biological factors, is a devastating complication of fracture repair. The most common causes of non-union include inappropriate stabilization, bone loss, infection, poor environment, systemic factors, and inadequate postoperative care (1, 2). Non-union can be classified as viable (hypertrophic, slightly hypertrophic, or oligotrophic) or non-viable (dystrophic, necrotic, defect, or atrophic) (2). Surgical intervention is the gold standard of care and is guided by the etiology of the non-union (2).

Among the various treatment options, bone grafting has been extensively applied in veterinary medicine. Autogenous cancellous bone graft is the most popular bone graft in clinical practice; it has optimal osteogenic, osteoinductive, and osteoconductive properties, with low material costs and no risk of host rejection (3). However, it is linked to limited availability in small or geriatric patients, morbidity at the donor site, and increased surgical times for graft retrieval (4). Allogenous cortical bone grafts from bone banks provide better mechanical stability for large bone defects, but their availability is sometimes limited. Autogenous rib grafts, which are corticocancellous, strong, and readily available, have been used in canine orthopedic surgery (4, 5). However, their application in felines has never been reported.

Bone morphogenetic proteins (BMPs) belong to the transforming growth factor superfamily and are most commonly used for their osteoinductive properties. Recombinant human bone morphogenetic protein (rhBMP)-2 and rhBMP-7 are beneficial for treating open fractures and non-unions in human medicine (6). In veterinary medicine, large bony defects treated with rhBMP-2 and scaffolds have achieved bone bridging. Few complications, including ectopic bone development and significant inflammation at the affected site, have been reported in dogs (7–10).

Very few clinical reports have been published regarding the use of fixation with autologous structural bone grafting and rhBMP-2 in cats (11–13). This article describes the successful treatment of non-union of a femoral fracture with a large defect using an autogenous rib bone graft and rhBMP-2 in a cat.

## Case Description

A 5-year-old, intact male Bengal cat weighing 5.2 kg (body condition score: 3/9) suffered a fracture of the right femur following a motor vehicle accident. A referring veterinary hospital performed surgical fixation using a dynamic compression plate, screws, and cerclage wires. Three weeks after surgery, the cat was presented to the Veterinary Medical Teaching Hospital at the National Pingtung University of Science and Technology due to the recurrence of non-weight-bearing right hind limb lameness.

**Figure 1:**
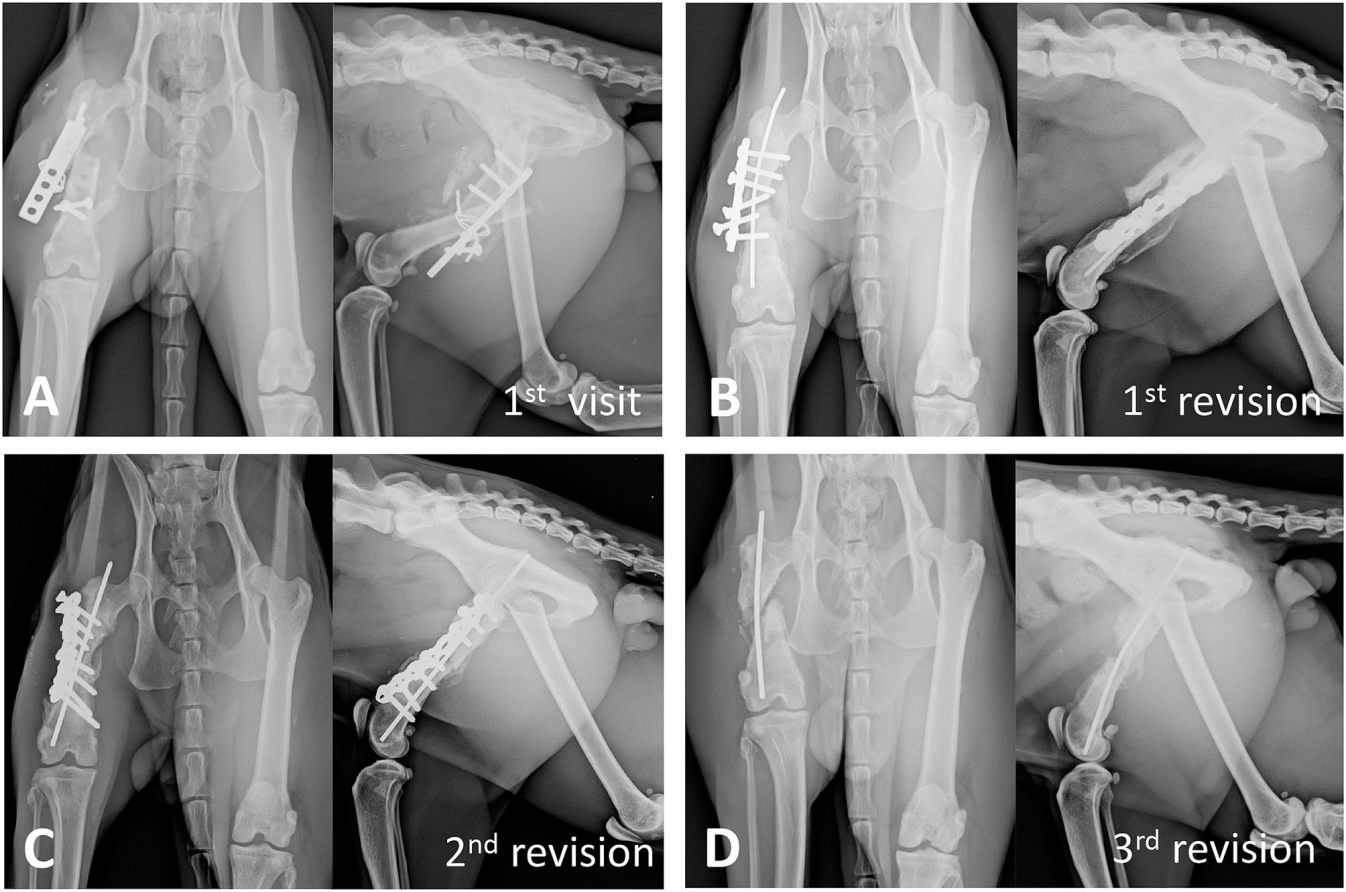
Radiographic progression of bone non-union. **(A)** At the first visit, radiographs of the right hind limb show a diaphyseal comminuted fracture of the right femur with active bone callus formation and implant failure. **(B)** Three months after the first revision surgery, the radiographs of the right femur show implant loosening, bone shortening, and no new callus formation in the fracture gap. **(C)** Four months after the second revision surgery, although autogenous cancellous bone graft was applied during the surgery, implant loosening and non-union are still noted. **(D)** After 11 months and multiple surgical revisions, significant atrophic non-union and bone shortening are noted.

Initial physical examination revealed pain and instability upon palpation of the right femur with soft tissue swelling. No other physical abnormalities were observed. Radiographic examination revealed a closed, comminuted, mid-diaphyseal fracture of the right femur with notable bone callus formation and implant failure (Figure 1A).

## Diagnostic Assessment, Therapeutic Interventions, and Outcomes

**Figure 2:**
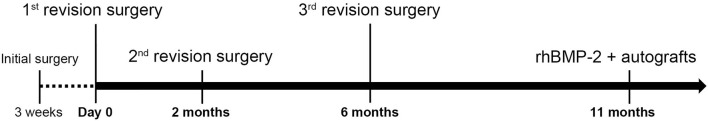
A brief timeline of the surgeries performed on the cat. The initial surgery was performed 3 weeks prior to the first revision surgery. The second and third revision surgeries were performed 2 months and 6 months after the first revision surgery, respectively. The final surgery applying rhBMP-2 and autografts was performed 11 months after the first revision surgery.

Three surgical revisions were performed before the final revision, along with concurrent activity restriction, analgesic medication administration, and low-level laser therapy. During the first revision surgery, the previous implants were removed and the fracture edges were debrided. The fracture was stabilized with a 2.7-mm non-locking reconstruction plate and a 1.6-mm intramedullary (IM) pin. After 2 months, the cat presented with persistent lameness and muscular atrophy of the affected limb on palpation. Radiographic examination indicated 30% femoral shortening and loosening of the four distal screws (Figure 1B). A second revision surgery was performed; the fracture edges were debrided, and an autologous cancellous bone graft harvested from the proximal ipsilateral humerus was placed around the fracture. The fracture was stabilized using a longer 2.7-mm plate and a 2.0-mm IM pin. However, 4 months later, the lameness remained unimproved, and radiographs showed delayed union with screw loosening (Figure 1C). Treatment options including femur amputation, type IA external skeletal fixation alone or combined with a tie-in technique, and autograft combined with BMP were discussed with the owner. In the meantime, the loosened reconstruction plate and screws were removed to minimize damage to the surrounding soft tissue and bone in a third revision surgery. The IM pin was left in place according to the owner's request although it provided only minimum bone fracture support. At presentation 11 months later, atrophic non-union of the affected femur was noted (Figure 1D). The owner eventually opted for fracture repair using a structural bone autograft and BMP. The surgical events of this case are shown in the timeline (Figure 2).

**Figure 3:**
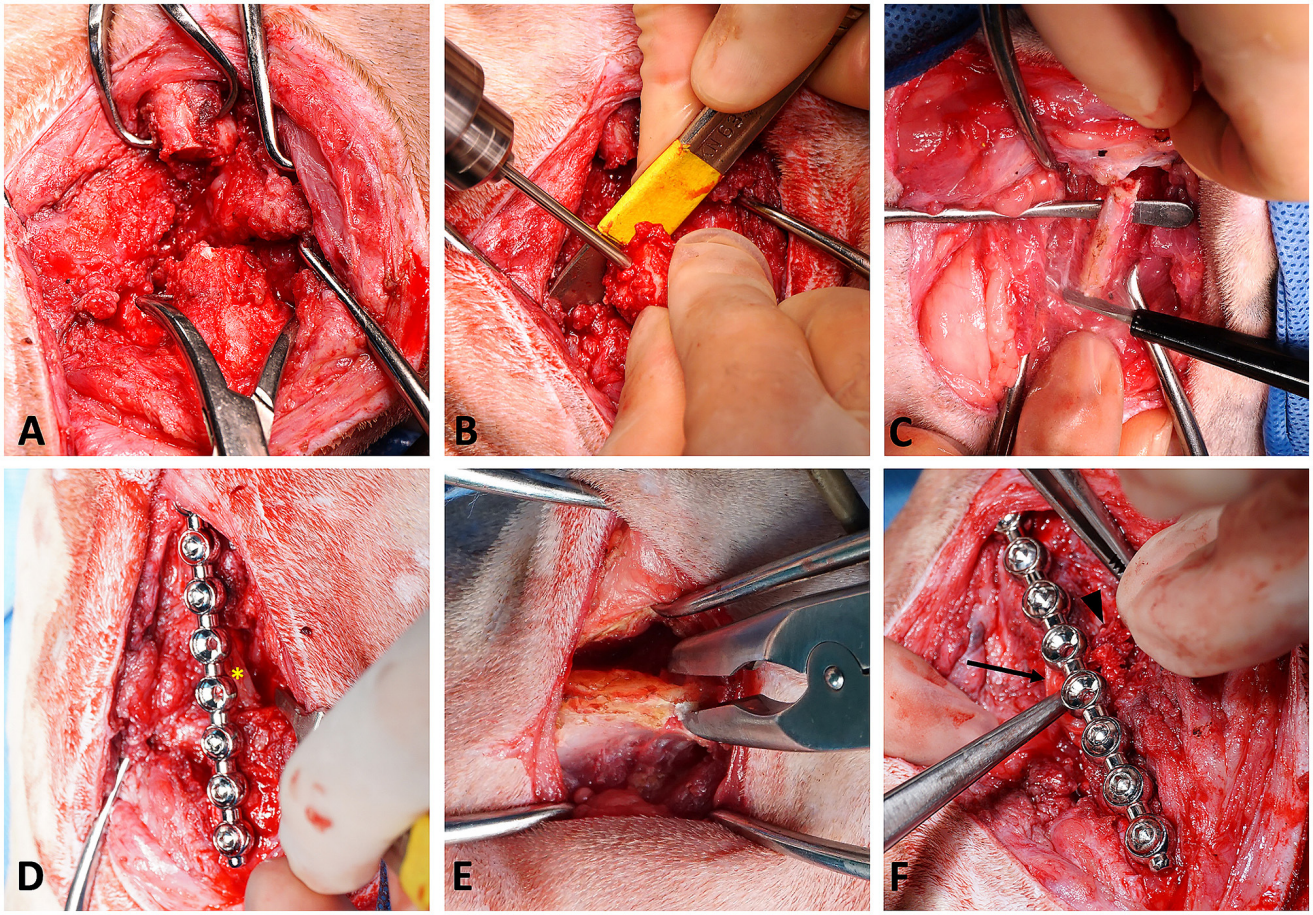
Surgical procedures. **(A)** Debridement of the diaphyseal fracture site of the right femur using a harmonic scalpel. **(B)** Medullary recanalization using a 2.8-mm pin. **(C)** Autograft harvested from the ipsilateral seventh rib. **(D)** Fixation using a locking plate system followed by transplantation of the autologous rib; the asterisk indicates the rib autograft. **(E)** Autograft harvested from the ipsilateral iliac crest. **(F)** Application of the rhBMP-2-soaked collagen sponge onto the site of defect; the arrow indicates the soaked sponge surrounding the bone defect, and the arrowhead indicates the corticocancellous iliac autograft.

The preoperative complete blood count and serum biochemistry results were within normal limits. Fentanyl (5 μg/kg) and cefazolin (22 mg/kg) were administered intravenously as a premedication and prophylactic antibiotic, respectively. General anesthesia was induced with intravenous propofol (6 mg/kg) and maintained with 1–2% inhaled isoflurane. Intraoperative analgesia was administered via a constant rate infusion of fentanyl (1.5 μg/kg/h), lidocaine (1.2 mg/kg/h), and ketamine (0.15 mg/kg/h). The cat was positioned in left lateral recumbency, and the previous IM pin was removed from the trochanteric fossa. Subsequently, the fracture site was exposed using a lateral approach in a routine manner. When fully exposed, the fracture edges were surrounded by smooth fibrotic tissue and mild callus formation was noted. The fracture edges were debrided with an ultrasonic surgical device (SonoCure, Tokyo Iken Co., Tokyo, Japan) (Figure 3A). The medullary canal was recanalized by drilling a 2.8-mm pin into the canal from the fracture site (Figure 3B). The fractured edges were maximally distracted using bone-holding forceps, although the original bone length could not be restored due to excessive soft tissue tension. An 8-hole, 66-mm, ball-shape reconstruction locking plate (Lisen Tech., Taoyuan City, Taiwan) with six 2.7-mm locking self-tapping cortical screws was used to stabilize the fracture segments; two holes in the middle were left empty. A bone gap of ~1.8 cm was measured in the defect.

**Figure 4:**
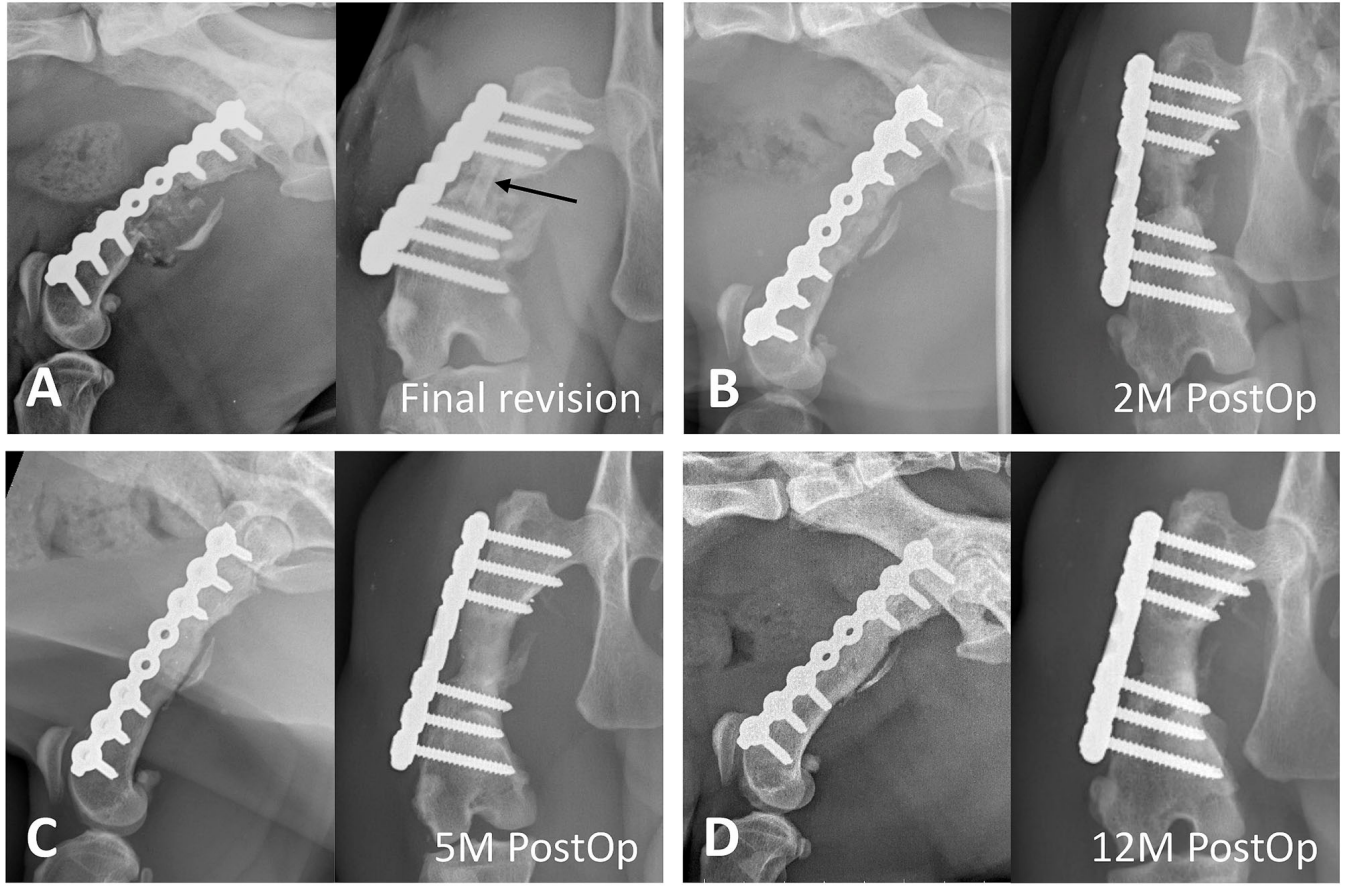
Radiographic progression of bone fracture repair. **(A)** Immediately after the final revision surgery, rib (arrow) and iliac crest autografts are implanted into the bone defect, and a locking plate is applied to stabilize the fracture. Mild distal femoral varus is noted. **(B)** At 2 months postoperatively, bridging of the callus and resorption of the rib graft are noted. **(C)** At 5 months postoperatively, the bone defect shows new bone formation, and the transplanted rib graft is indiscernible. **(D)** At 12 months postoperatively, remodeling and maturity of the regenerated bone are seen.

The ipsilateral seventh rib was approached by making an incision through the skin and thoracic muscles. The periosteum of the rib was incised to expose the mid-section of the rib body, and it was elevated carefully. A 2-cm segment of the rib was harvested using an ultrasonic scalpel (SonoCure, Tokyo Iken Co., Tokyo, Japan) (Figure 3C). The intercostal muscles were then apposed over the cut edge of the rib with 3-0 polydioxanone suture in a simple interrupted pattern. The length of the graft was chosen to be the length of the fracture gap plus 0.5 mm. The graft was then positioned at the center of the fracture gap with gentle manual impaction (Figure 3D). No additional fixation was performed. Another skin incision was made over the ipsilateral iliac crest. Several pieces of corticocancellous iliac autograft were obtained with a rongeur (Figure 3E) and placed to fill the residual space around the rib graft. Finally, a 3 ×5 cm absorbable collagen sponge (SavPet, Oriental Co., Taipei, Taiwan) was soaked in 100 μg of rhBMP-2 (10426-HNAE, Sino Biological, Beijing, China) reconstituted in 0.5 mL of normal saline and wrapped around the bone defect (Figure 3F). Routine closure of the three surgical sites was performed. As the pleura was left intact, a thoracostomy tube was not required. Although the exact range of motion was not measured by a goniometer during surgery, the range of motion of the right stifle was subjectively judged to be about half that of the contralateral side on physical exam before extubation. Postoperative radiographs were obtained to confirm the position of the graft (Figure 4A). Mild distal femoral varus was also noted (Figure 4A). The radiographically measured length of the right femur was ~8.0 cm, and that of the left femur was 12.1 cm (~34% shortening).

Anesthetic recovery was uneventful. Pain was managed with intravenous tramadol (2 mg/kg) twice a day for 2 days, a single dose of intravenous meloxicam (0.1 mg/kg), and low-level laser therapy on the surgical sites. Intraoperative aerobic and anaerobic bacteria cultures were performed on a sample collected from the bone defect before debridement, and no pathogens were identified. No postoperative complications were observed. The cat was discharged on the fourth postoperative day with an instruction of cage rest.

On the seventh postoperative day, occasional weight-bearing lameness during walking was noticed (lameness score: 4/5) (14). After 2 months, the cat was able to trot with mild lameness at home. On physical examination, the cat was consistently bearing weight on the affected limb with a moderate weight-bearing lameness during walking (lameness score: 3/5) characterized by a decreased swing phase. Radiographs showed bridging callus formation with new bone formation and incorporation of the transplanted rib graft (Figure 4B). On radiographs taken 5 months postoperatively, bony union at the fracture gap was evident and the transplanted rib graft was indiscernible (Figure 4C). Moreover, at the last recheck examination 12 months postoperatively, successful bone remodeling and apparent maturity of the generated bone were noted (Figure 4D). To compensate for the discrepancy in hind limb length, the cat stood with the affected hip, stifle, and hock joints at greater angles of extension. Despite mild lameness during walking (lameness score: 2/5) (see Supplementary Video 1), the cat was energetic and had no difficulty jumping at home. The passive range of motion of the right stifle was also increased but was still about 30% less than contralateral side on physical exam without application of anesthesia or sedation. The owner was satisfied with the improved health status and good ambulatory function.

## Discussion

The treatment of a femoral non-union with a diaphyseal defect using rib and iliac autografts in combination with the growth factor rhBMP-2 was successful in this case. Radiographic examination revealed new bone formation 2 months postoperatively and a progressive increase in the bone union 5 months postoperatively, with no complications noted in the 1st year after surgery. Although mild mechanical weight-bearing lameness persisted, the cat's ambulatory function and quality of life were good.

In cats, the prevalence of non-union in long bone fracture has previously been reported to be 0.85–4.3% (15, 16). Specific anatomical regions known to have a higher incidence of non-union are the tibia/fibula followed by the radius/ulna, with the rate of non-union reaching as high as 15% in the tibia/fibula, 5% in the radius/ulna, and 1% in the femur (16). Non-union fracture in the femur is less likely to develop in cats because of the abundant tissue covering the femur. Risk factors in cats include older age, higher body weight, affected bone, fracture type, degree of comminution, and fixation type (16). In this case, the risk factors for development of the non-union included fracture comminution and inadequate mechanical environment due to the fixation methods in the initial and subsequent revision surgeries: insufficient number of screws placed on the distal bone fragment with a non-locking plate in the initial surgery; the plate was too short and a small diameter of IM pin was used in the first and second revision surgeries; only an IM pin was left in site in the third revision surgery. In addition, the retrograde IM pin was too long and protruded at the proximal end of the femur, which predisposed the cat for sciatic nerve injury. Finally, greater bone loss caused by aggressive debridement with surgical trauma to the surrounding tissue may have contributed to the atrophic non-union before the final revision.

A locking plate was used for fixation in the final revision surgery. A locking plate is advantageous to a non-locking reconstruction plate because it allows for a more stable fixation, with increased resistance to bending over the large fracture gap while still providing adequate strain to promote bone formation (17).

To minimize limb shortening during the final surgery, the fracture segments were distracted as much as the soft tissues would allow. This distraction subsequently created a significant bony defect. Critical bone defects, which represent a significant challenge in both human and veterinary medicine, do not heal spontaneously (18). Traditionally, allograft bone grafting with supporting and conduction effects is primarily used (18). However, the disadvantages of commercial allografts include limited availability, higher costs, and risk of host rejection, and therefore, the use of autogenous bone grafts is preferred to overcome these disadvantages. In this case, a segmental autogenous cortical graft from the contralateral femur was considered to provide structural support and increase the length of the right femur. However, the potential risk of morbidity at the contralateral femoral diaphysis, which could lead to a second non-union event, was a concern. Therefore, a segment of the seventh rib was selected due to its size and surgeon preference. Furthermore, accessibility was considered easier with less surgical trauma than that of the femur. Additionally, an iliac autograft was used adjunctively to fill the remaining space and provide an osteogenic effect, rather than mechanical support.

A cancellous autograft acquired from the humerus was used for the initial surgical revisions in this case; however, fracture union still failed to occur, and substantial bone loss occurred due to frequent debridement. A previous study showed that using rhBMP-2 with an absorbable collagen sponge provided faster bone union than a bone graft alone in dogs (19). A benefit of rhBMP-2 is enhanced angiogenesis, which is particularly helpful in cases with vascular impairment from multiple surgeries (2, 7). In this case, we used an absorbable collagen sponge as a delivery agent loaded with 0.5 mL of 0.2 mg/mL rhBMP-2 solution. The concentration and dose of rhBMP-2 were based on published clinical reports (10, 11, 20). The total dose of rhBMP-2 (100 μg) was lower than those used in experimental trials (19, 21). According to our results, adequate bone union was achieved, and the reported adverse effect of ectopic/cystic bone formation was not noted (7, 8). The autogenous rib and iliac bone grafts placed in the fracture gap may have also reduced the required dose of rhBMP-2, leading to cost reduction.

Dogs can tolerate up to 20% of femur shortening without exhibiting lameness by adjusting the joint angles bilaterally (22). The maximum amount of limb discrepancy tolerated in cats without producing lameness has not yet been reported. However, the aforementioned data could still be extrapolated due to the similarity in the skeletal frames of dogs and cats. In this case, the final revision surgery resulted in 34% femoral shortening, and postoperative lameness was expected with this degree of discrepancy. The rib graft contributed to 15% of the femoral length and presumably reduced the severity of lameness. Although a longer rib graft could have increased femoral length and potentially reduced lameness, the contracture of the thigh muscles limited further traction of the bone fragments. Additionally, the femoral varus and reduced range of motion in the stifle may have contributed to the lameness.

## Conclusion

The combined use of rib and iliac autografts and rhBMP-2 provided a feasible treatment option for the management of non-union with a large bone defect in a femoral fracture in this case without complications. The advantages of this procedure include reducing the length discrepancy of the hind limbs, constructing a stronger mechanical support for the fixation frame, providing a potent osteoinductive environment, and reducing the required dose of expensive growth factors. To the best of our knowledge, this is the first report of the clinical application of a segmental rib as a corticocancellous autograft combined with rhBMP-2 in a cat. However, as an individual case study, the contraindications and complications of rib grafts with rhBMP-2 in large bone defects in cats cannot be fully confirmed. Further, the optimal dose of rhBMP-2 for cats was not determined here, and more clinical trials are warranted to further evaluate this procedure in other clinical settings.

## Data Availability Statement

The original contributions presented in the study are included in the article/Supplementary Material, further inquiries can be directed to the corresponding author/s.

## Author Contributions

LSL and YMT collected the data and edited the manuscript. CSC performed all of the surgeries, contributed to the study conception, and critically revised the manuscript. All authors contributed substantially to the data interpretation and final approval of the manuscript.

## Funding

This research was supported by the Southern Taiwan Science Park Bureau, Ministry of Science and Technology, Taiwan, R.O.C. under contract 110CB-1-04.

## Conflict of Interest

The authors declare that the research was conducted in the absence of any commercial or financial relationships that could be construed as a potential conflict of interest.

## Publisher's Note

All claims expressed in this article are solely those of the authors and do not necessarily represent those of their affiliated organizations, or those of the publisher, the editors and the reviewers. Any product that may be evaluated in this article, or claim that may be made by its manufacturer, is not guaranteed or endorsed by the publisher.

## Supplementary Material

Supplementary Video 1 At the recheck appointment 12 months after the final revision surgery, although mild lameness persists, the ambulatory function is good.

## Abbreviations

BMP: bone morphogenetic protein
IM: intramedullary
rhBMP: recombinant human bone morphogenetic protein.
